# Protecting mother and baby: Learnings from an Ebola vaccination campaign on the evolving landscape of vaccines and pregnancy

**DOI:** 10.1371/journal.pmed.1004530

**Published:** 2025-02-11

**Authors:** Rebecca F. Grais, Kathleen M. Neuzil, Helen Rees, Cristiana M. Toscano

**Affiliations:** 1 Pasteur Network, Paris, France; 2 Fogarty International Center, National Institutes of Health, Bethesda, Maryland, United States of America; 3 University of Witwatersrand, Johannesburg, South Africa; 4 Institute of Tropical Pathology and Public Health, Federal University of Goiás, Goiânia, Brazil

## Abstract

This Perspective article from Rebecca Grais and colleagues places a recently published study in PLOS Medicine in the context of the evolving landscape of vaccines and pregnancy.

For women of childbearing age or those planning to become pregnant, vaccination recommendations vary depending on risk of disease exposure and severity, presence of health conditions or co-morbidities, and the type of vaccines available. Compared to nonpregnant individuals, data on vaccines and vaccination in pregnancy is severely limited on a global scale. One of the key reasons for this is the lack of inclusion of pregnant women in vaccine development trials. The reasons for this exclusion traces back for decades and has a complex history, driven by ethical and safety concerns, cultural norms, scientific uncertainty, and regulatory practices, among others. Historically, vaccine studies have followed a “wait-and-see” approach, where a vaccine would be widely studied and administered to the general population first. Pregnant women were excluded until the safety profile was well-established in other groups, and evaluation of safety and effectiveness data on pregnant women’s responses to vaccines was generally deferred to post-marketing surveillance, where data from inadvertent vaccinations or observational studies of vaccinated populations were used to generate evidence. Many regulatory bodies, including the US Food and Drug Administration (FDA) and the European Medicines Agency (EMA), adopted policies that were conservative about the inclusion of pregnant women in trials, preferring to focus on fetal safety and avoid potential risks [1].

This approach began to change with the emergence of infectious diseases with high maternal and infant morbidity and mortality. In recent epidemics, such as Ebola and COVID-19, for example, exposure to disease was often greater among women of childbearing age than among the general population, as women are overrepresented among health care workers and family care givers [2]. Thus, the exclusion of pregnant women and their lack of access to potentially life-saving vaccines came under ethical scrutiny. To address the evidence gaps and inequities, multiple stakeholders, including the World Health Organization (WHO), challenged the current norms and advocated for the inclusion of pregnant women early on in vaccine trials [3]. Regulatory agencies have updated their policies to better accommodate the inclusion of pregnant women in clinical research, particularly for vaccines with a clear need, such as during infectious disease outbreaks with limited preventive or therapeutic alternatives available [4]. Frameworks have been developed that allow for the ethical inclusion of pregnant women in vaccine trials, including phased approaches, standardized and systematic monitoring of adverse events, risk assessment of different investigational products if used in pregnancy and separate consent processes tailored to the unique risks in pregnancy [5]. Global institutions have advocated for the responsible inclusion of pregnant women in clinical trials. They emphasize that lack of data can lead to even greater harm by leaving pregnant women vulnerable during outbreaks. This shift in approach emphasizes the importance of beginning from a “presumption of inclusion”—providing information on the risk and benefits and then allowing pregnant women to make their own decisions on enrolling in a trial or receiving a vaccine [6].

One of these infectious vaccine-preventable diseases is Ebola. Studies have reported maternal mortality rates ranging from approximately 68% to over 90% [7]. Neither of the 2 currently licensed and WHO-prequalified vaccines against Ebola included pregnant women in clinical development trials, although the vaccine has been used post-licensure.

Between 2019 and 2021, a two-dose Ebola virus vaccine was offered to all Rwandans aged 2 years and above, at risk of Ebola virus disease. Pregnant women were excluded from the campaign. An outbreak of Ebola virus in neighboring Democratic Republic of Congo increased risk for the population. As a part of the vaccination campaign, women who were fertile and sexually active were counseled about preventing pregnancy and offered contraception.

In a new study, Rosine Ingabire and colleagues report on the use of contraception, pregnancy incidence, and serious adverse events (SAEs) collected during the vaccination campaign [8]. Of the 47,585 fertile and sexually active women participating in the campaign, two-thirds were not using contraception at baseline (prior to dose one). The study team detected 969 incident pregnancies after dose one, of which 19% resulted in an obstetric SAE with almost all ending in pregnancy loss. Available data suggest these percentages are in line with a previous estimate from Rwanda of 16% [8]. No SAE were determined to be related to vaccine. Whereas a majority (95%) of women without a pregnancy went on to receive the second vaccine dose, only 34% of women who became pregnant after the first vaccine dose received the second dose after pregnancy completion.

The authors conclude that contraception should be offered during two-dose vaccination regimens to ensure maximum protection. While the Ingabire and colleagues report offers insights into the specific context in Rwanda and prevention of Ebola, many of the challenges and opportunities are mirrored elsewhere [9]. Variation in local cultural, economic, and health system indicate that strategies must be adapted accordingly to be effective on a broader scale. First, in many communities, including parts of Rwanda, cultural and religious beliefs can pose a significant barrier [10]. Discussions around contraception can be controversial, as many women face societal and familial pressure to avoid contraceptive use due to perceptions that it promotes promiscuity or undermines traditional values. Religious opposition to contraception can make women hesitant to access vaccines linked to contraception requirements. This stigma can be intensified when contraception is a precondition for vaccination. Second, lack of access to comprehensive sexual and reproductive health education makes it difficult for women to make informed decisions regarding contraception. Additionally, limited access to healthcare, particularly in rural areas, may result in absence of regular consultations with healthcare professionals during which both vaccination and contraception could be discussed [11]. Health workers may also lack training on how to effectively communicate the need for vaccination in tandem with contraceptive use. Third, some women may fear side effects from both vaccines and contraceptives, leading to reluctance in participation. Fourth, policies requiring mandatory contraception before vaccination can create unnecessary bureaucratic hurdles. For instance, some women may lack access to contraceptives or may not be comfortable with using certain forms of birth control, which limits their ability to access life-saving vaccines. Furthermore, issues such as supply chain challenges for both vaccines and contraceptives can impede service delivery.

These barriers point to clear opportunities to address challenges for vaccine delivery as well as contraception. Communication plans and community engagement are essential. Offering integrated reproductive health services that combine vaccine provision with access to contraception can improve outcomes. In Rwanda, programs that bring multiple health services together in one setting have shown promise in increasing access to both vaccines and contraceptives [12]. Government policies that support women’s autonomy in making health decisions can lead to improved uptake of both vaccines and contraceptives. Creating flexible policies that do not require mandatory contraception but encourage informed choice may also improve outcomes. New developments in vaccine and contraceptive technologies (such as long-acting reversible contraceptives) can make it easier for women to manage both their reproductive health and immunization schedules. Mobile health technology can also support women by providing them with reminders and information about vaccination and contraceptives. In the end, the goal is to maximize health benefits for pregnant women and their offspring.
